# Identification of Valerate as Carrying Capacity Modulator by Analyzing *Lactiplantibacillus plantarum* Colonization of Colonic Microbiota *in vitro*

**DOI:** 10.3389/fmicb.2022.910609

**Published:** 2022-05-30

**Authors:** Julia Isenring, Marc J. A. Stevens, Christoph Jans, Christophe Lacroix, Annelies Geirnaert

**Affiliations:** ^1^Laboratory of Food Biotechnology, Department of Health Sciences and Technology, ETH Zürich, Zürich, Switzerland; ^2^Institute for Food Hygiene and Safety, University of Zürich, Zürich, Switzerland

**Keywords:** gut microbiome, probiotics, intestinal ecology, PolyFermS, microbiota invasion

## Abstract

Humans ingest many microorganisms, which may colonize and interact with the resident gut microbiota. However, extensive knowledge about host-independent microbe-microbe interactions is lacking. Here, we investigated such colonization process using a derivative of the model probiotic *Lactiplantibacillus plantarum* WCFS1 into continuously cultivated gut microbiota in the intestinal PolyFermS fermentation model inoculated with five independently immobilized human adult fecal microbiota. *L. plantarum* successfully colonized and organized itself spatially in the planktonic, that is, the reactor effluent, and sessile, that is, reactor biofilm, fractions of distinct human adult microbiota. The microbiota carrying capacity for *L. plantarum* was independent of *L. plantarum* introduction dose and second supplementation. Adult microbiota (*n* = 3) dominated by *Prevotella* and *Ruminoccocus* exhibited a higher carrying capacity than microbiota (*n* = 2) dominated by *Bacteroides* with 10^5^ and 10^3^ CFU/ml of *L. plantarum*, respectively. Cultivation of human adult microbiota over 3 months resulted in decreased carrying capacity and correlated positively with richness and evenness, suggesting enhanced resistance toward colonizers. Our analyses ultimately allowed us to identify the fermentation metabolite valerate as a modulator to increase the carrying capacity in a microbiota-independent manner. In conclusion, by uncoupling microbe-microbe interactions from host factors, we showed that *L. plantarum* colonizes the *in vitro* colonic community in a microbiota-dependent manner. We were further able to demonstrate that *L. plantarum* colonization levels were not susceptible to the introduction parameters dose and repeated administration but to microbiota features. Such knowledge is relevant in gaining a deeper ecological understanding of colonizer-microbiota interactions and developing robust probiotic strategies.

## Introduction

The amount of research linking distortion of the gut microbiota composition and functionality to diseases led to the arise of gut microbiota modulation strategies such as probiotics, consortia, or fecal microbiota transplantation (Wargo, 2020). Although probiotics belong to the most frequently used approaches, underlying modes of action are still poorly understood and probiotic effectiveness shows a high individual variation (Ojima et al., 2022). It is therefore of interest to deepen our ecological knowledge about how ingested strains may behave, colonize, and impact the resident colonic microbiota.

Colonization of the gut microbiota by an exogenous MO is influenced by the host, the colonizer, and the resident community. Host physiology, metabolism, and immune system are factors influencing the colonization process (Mallon et al., 2015). The host further provides attachment sites and resources like the mucus layer (Frese et al., 2013). Colonizers' traits linked to the successful establishment in the community are adaptability, phenotypic plasticity, growth rate, and competitiveness (Nyberg and Wallentinus, 2005; Catford et al., 2009; Mallon et al., 2015; Tao et al., 2018; Walter et al., 2018). The dose and intake frequency of the exogenous strain might further impact colonization success (Marco and Tachon, 2013). Also, the resident microbiota highly influences colonization outcomes. First, the microbiota exhibits a carrying capacity, defined as the maximum count of an individual member that is supported by a specific environment, and second, colonization resistance, which refers to the potential of the resident community to prevent colonization (McArthur, 2006). Colonization resistance is desired to avert pathogen infections but undesired for probiotics (Vollaard and Clasener, 1994; He et al., 2014).

Colonization resistance mechanisms comprised antimicrobial production, nutrient competition, or bacteriophage development (Vandenbergh, 1993; Ducarmon et al., 2019). Colonization resistance correlates positively with community richness and diversity by increasing the likelihood of harboring an efficient competitive taxa over the colonizer (Shea and Chesson, 2002; Dunstan and Johnson, 2006; Jousset et al., 2011; Kinnunen et al., 2016) and by using resources more efficiently (van Elsas et al., 2012; Mallon et al., 2015). However, these concepts are rarely experimentally validated in the human gut microbiota. Moreover, the interdependence of the host, colonizer, and microbiota impede the identification of single determining microbiota factors. Well-validated *in vitro* gut fermentation microbiota models exclude the host factor and hence allow solely studying colonizer-microbiota interactions. The continuous PolyFermS colonic fermentation model, inoculated with immobilized fecal microbiota mimicking planktonic and sessile cell growth, was recently successfully established for adaptive evolution of *Lactiplantibacillus plantarum* in human adult colonic microbiota (Isenring et al., 2021b). Immobilization of fecal microbiota prevents wash-out during continuous operation, resulting in a long-term stable, high-cell-density community (Lacroix et al., 2015). Therefore, this system may be well-suited to investigate colonizer-microbiota interactions and therefore deepen our understanding of the ecological fate of an exogenous strain in the gut microbiota.

Here, we investigated the colonization process of *L. plantarum* into different human adult colonic microbiota using the validated continuous PolyFermS model (Isenring et al., 2021b). *L. plantarum* is a versatile lactic acid bacterium that survives gastric and intestinal stress conditions and is thus able to arrive in the intestine in high levels and viable forms (Vesa et al., 2000; Pavan et al., 2003; Mohedano et al., 2019). Therefore, we used *L. plantarum* NZ3400, a derivative of the model probiotic WCFS1, harboring an antibiotic resistance gene integrated into the chromosome that allows selective tracing within the colonic community to characterize its colonization and identify community characteristics involved in this process.

## Materials and Methods

### Bacterial Strains and Growth Conditions

*L. plantarum* NZ3400 is a WCFS1 derivative strain harboring chloramphenicol (CM) resistance cassette on a neutral locus of the genome that allows tracking of the strain (Remus et al., 2012). A single colony of NZ3400, designated NZ3400B, was used in this study to investigate the colonization of *in vitro* colonic microbiota (Isenring et al., 2021b). Further, *L. plantarum* NZ3400B derivatives IA10 (C569A in LP_RS14255), PA2_06 (C837A in LP_RS15205), and PA1.2_01 (C979T in LP_RS14990 and G382A in LP_RS01530), all recovered from colonic microbiota after long-term cultivation, and an LP_RS14990 gene replacement *L. plantarum* NZ3400B (ΔLP_RS14990) were used (Isenring et al., 2021b). Strains were cultivated in De Man, Rogosa, and Sharpe (MRS, Labo-Life Sàrl, Switzerland) broth at 37°C. Enumeration of viable cells was done by plating on CM (10 mg/ml) supplemented MRS agar (MRS + CM), aerobically at 37°C, overnight.

MacFarlane medium mimicking the chyme entering the proximal colon was used for the cultivation of adult colonic microbiota (Isenring et al., 2021b). The heat-sterilized nutritive medium was supplemented with filter-sterilized vitamin solution (Michel et al., 1998).

### Immobilization of Donor Fecal and Cecum Microbiota

**Figure 1:**
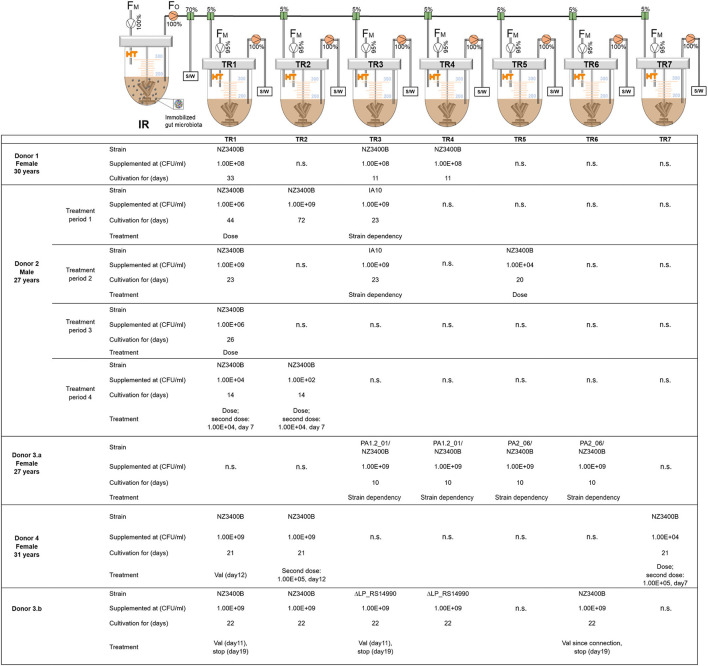
Setup to investigate colonization of *L. plantarum* in human adult *in vitro* colonic microbiota [adapted from (Isenring et al., 2021b)]. The setup was repeated five times with the inoculum reactor (IR) inoculated with fecal microbiota obtained from four different donors (1–4) and donor 3 being repeated using two fecal samples collected 6 months apart. The IR effluent was used to continuously inoculate (5% effluent and 95% fresh medium feed rate) second-stage test reactors (TRs), providing independent replicates when supplemented in parallel with the same colonizer strain. TRs were supplemented with *L. plantarum* at different doses with different strains. NZ3400B, single colony isolates of NZ3400; IA10, PA1.2_01, PA2_06, *L. plantarum* NZ3400B mutants isolated from *in vitro* colonic microbiota; *L. plantarum* ΔLP_RS14990, LP_RS14990 gene deletion strain; F_M_, Inflow MacFarlane medium; F_O_, Reactor outflow; S/W, Sampling/Waste; Val, Valerate; n.s., reactors applied with other experimental conditions that are not included in this study.

Fecal samples of four healthy adults (27–31 years), who did not receive antibiotics and probiotics for 3 months before donation, were collected. Samples were screened for the absence of detectable growth on MRS+CM agar to ensure selective tracking of *L. plantarum* NZ3400B. The study was exempted from review by the Ethics Committee of ETH Zürich due to the absence of intervention during sample collection. Fresh fecal samples were transferred into an anaerobic chamber. About 5 g of fecal sample were homogenized in 25 ml reduced peptone water (0.1%, pH = 7; Thermo Fisher Diagnostics AG, Pratteln, Switzerland), resulting in 20% (w/v) fecal slurry which was used for immobilization as described previously (Cinquin et al., 2004; Fehlbaum et al., 2015) to entrap the fecal microbiota in polymer gel beads (gellan gum (2.5%, w/v), xanthan (0.25%, w/v), and sodium citrate (0.2%, w/v)). And 60 ml of fecal beads were used to inoculate the inoculum reactor (IR, Figure 1) (Sixfors, Infors, Bottmingen, Switzerland), filled with 140 ml of nutritive medium, followed by two repeated batch fermentations operated at stirring at 180 rpm, 37°C, and pH = 5.8 by addition of NaOH (2.5 M), resulting in colonized beads. Between batch fermentations, a 100 ml fermented medium was replaced with a fresh medium after 16 h (Isenring et al., 2021b).

### Operation of Continuous Microbiota Fermentation

Immobilized fecal microbiota were cultivated continuously in the IR with a volume of 200 ml, run at 37°C, pH = 5.8, and stirred at 180 rpm to simulate the proximal colon environment (Berner et al., 2013; Tanner et al., 2014a). A fresh nutritive medium was added to the bead-containing IR at 25 ml/h, resulting in a retention time of 8 h. The reactor's headspace was purged with CO_2_ to create and maintain anaerobiosis. A < 10% day-to-day variation in fermentation metabolites was set as a cut-off for metabolic stability (Isenring et al., 2021b). After reaching stable fermentation, second-stage treatment parallel reactors (TRs) were connected to the IR (Figure 1). TRs were operated with the parameters specified above for the IR and continuously inoculated with IR effluent (5% of the feed rate) and fed with fresh nutritive medium (95% of the feed rate). TRs were operated on for at least 4 days to achieve metabolic stability before *L. plantarum* supplementation.

To monitor the *in vitro* microbiota fermentation, 2 ml effluent samples were taken daily and centrifuged (10 min, 14'000 g, and 4°C). Supernatants were immediately used for metabolite analysis and the pellet was stored at −20°C for ~1 year until DNA extraction.

### Experimental Set-Up

Colonization of *L. plantarum* was investigated in four different *in vitro* adult colon microbiota and twice in the microbiota of donor 3 using samples acquired within half a year, resulting in donor 3.a and 3.b (Figure 1). For inoculation into TRs, *L. plantarum* was grown overnight and optical density was measured at 600 nm (OD_600nm_) using a spectrophotometer (Eppendorf, Hamburg, Germany). Cultures were set to the desired OD_600nm_ (1 ≈ 3^*^10^8^ CFU/ml), washed twice in PBS, and resuspended in the nutritive medium of the corresponding modeled host. For the general set-up, a single dose of 10^11^ CFU *L. plantarum* was added to TRs, containing 200 ml effluent, to reach 10^9^ CFU/ml, which corresponds to the dose used in previous *in vivo* trials (Costa et al., 2014; Lee et al., 2021).

In the case of several treatment periods within one microbiota, TRs were disconnected, sterilized, reconnected, and stabilized for at least 4 days before the next treatment period. The treatment period is included at the end of the reactor label, for example, TR1 during treatment period 2 corresponds to TR1.2.

#### Influence of Strain Dependency on *L. plantarum* Colonization

To investigate the influence of strain dependency on *L. plantarum* microbiota colonization, strain IA10 was compared to NZ3400B in the colonic microbiota of donor 2 (*n* = 2). A mix of two strains, PA1.2_01/NZ3400B and PA2_06/NZ3400B, was added (1:1 ratio) to the colonic microbiota of donor 3.a (*n* = 2). Furthermore, the LP_RS14990 gene replacement strain ΔLP_RS14990 was used in the microbiota of donor 3.b (*n* = 2) (Figure 1).

#### Influence of Supplementation Dose on *L. plantarum* Colonization

To investigate the influence of introduction dose on colonization, *L. plantarum* was added to the TRs containing donor 2 microbiota to reach the final concentrations of 10^9^ (*n* = 3), 10^6^ (*n* = 2), 10^4^ (*n* = 2), and 10^2^ CFU/ml (*n* = 1). We also tested supplementation of *L. plantarum* in TRs containing donor 4 microbiota, at final concentrations of 10^9^ (*n* = 2) and 10^4^ CFU/ml (*n* = 1) (Figure 1). The influence of a second *L. plantarum* dose was tested after stable colonization upon the first introduction in the microbiota of donor 2 and 4.

#### Influence of Microbiota Maturity on Carrying Capacity for *L. plantarum*

The microbiota of donor 2 was continuously cultivated for 3 months and TRs were connected and supplemented with *L. plantarum* at different time points (Figure 1). The increase in microbiota fermentation age will be further referred to as microbiota maturity.

#### Modulation of Microbiota Carrying Capacity by Valerate

The potential of valerate to modulate the microbiota carrying capacity for *L. plantarum* was investigated by adding it to the fresh nutritive medium after stable colonization by NZ3400B at concentrations relevant to the human intestine (McDonald et al., 2018). Valerate was added to obtain 12 mM in the microbiota of donor 4 (TR1) on day one and 6 mM in the following 6 days. A control reactor was not feasible to operate due to technical limitations. In donor 3.b colonic microbiota, valerate was added to obtain 12 mM in the reactor (TR1, TR3) (Figure 1), while TR2 and TR4 served as control reactor. Valerate supplementation was stopped after 8 days to investigate the impact on colonization level. Valerate was further added at 12 mM to TR6 after connection to determine the effect of valerate on colonic microbiota in absence of *L. plantarum* NZ3400B.

#### Monitoring of the Colonization Process of *L. plantarum* in *in vitro* Colonic Microbiota

*L. plantarum* colonization of five independent *in vitro* colonic microbiota was monitored by daily plating reactor effluent on MRS+CM agar. Naturally formed biofilms on the reactor's walls were collected at the end of fermentation by emptying and washing the reactor vessel twice with PBS to remove non-adherent cells. The complete biofilm was removed using a spatula and homogenized in falcon tubes containing dilution solution (0.85% NaCl, 0.1% peptone from casein (w/v), VWR International AG, Dietikon, Switzerland) using glass beads (5 mm, VWR International AG, Dietikon, Switzerland). *L. plantarum* viable cell count was determined by plating as described earlier.

### Microbial Metabolite Analysis by HPLC-IR

Microbiota activity and stability were monitored daily *via* SCFA analysis of the supernatants from reactor effluent samples. Concentrations of the main SCFAs end products (acetate, propionate, butyrate, and valerate), branched-chain fatty acids (isobutyrate and isovalerate), and intermediate metabolites (lactate, succinate, and formate) were measured by high-performance liquid chromatography as described previously (Tanner et al., 2014b).

### Microbial Community Analysis With 16S rRNA Gene Amplicon Sequencing

Total genomic DNA of fecal samples was extracted from the fecal slurry used for immobilization and from reactor microbiota from effluent pellets using the FastDNA® SPIN Kit for Soil (MP Biomedicals, Illkirch, France) according to the manufacturer's instructions.

Total 16S rRNA gene copy numbers were determined by quantitative real-time PCR (qPCR) using the Eub_339F (ACTCCTACGGGAGGCAG) and Eub_518R (ATTACCGCGGCTGCTGG) primer (Guo et al., 2008) as described previously (Bircher et al., 2020).

Microbiota analysis was done by sequencing the V4 region of the 16S rRNA gene using an in-house protocol as described previously (Isenring et al., 2021b). Library preparation, pooling, and sequencing (Illumina, CA, USA) were done in collaboration with the Genetic Diversity Center (GDC, ETH Zürich). Sequencing was performed on the Illumina MiSeq platform using a flow cell with V2 2x250-bp paired-end chemistry supplemented with PhiX [10% (v/v)].

Raw data processing into amplicon sequences variants (ASVs) and ASV taxonomy assignment were done as described previously (Isenring et al., 2021b).

### Data Analysis

Microbiota community analysis was done on rarefied samples in R (version 3.6.2) using the phyloseq package. The DESeq method was applied to non-rarefied data (Love et al., 2014; McMurdie and Holmes, 2014) to detect significant differences in ASV abundance between reactors containing the same colonic microbiota. To decrease the bias occurring due to ASVs that are abundant in only a few samples, only ASVs that contained more than 10 reads in more than 50% of the samples were kept for the DESeq analysis. All correlation analyses were done using Spearman correlation except for the correlation between carrying capacity and metabolite concentration using Pearson correlation.

Depicted community composition and metabolic profiles comprise the average and standard deviation of samples taken on 3 consecutive days. Graphs were created using GraphPad Prism® version 8 (GraphPad Software Inc., San Diego, CA, USA).

## Results

### *L. plantarum* Colonizes Different Human Adult Microbiota *in vitro*

**Figure 2:**
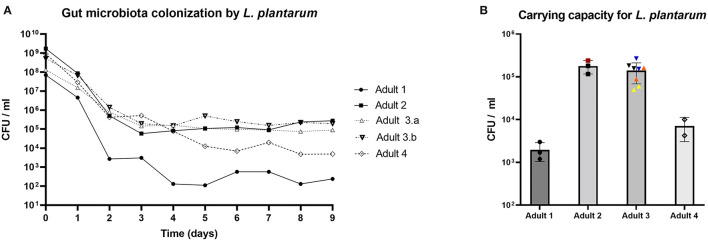
Colonization levels of *L. plantarum* in representative TRs connected during the first treatment period to the IR seeded with immobilized human adult fecal microbiota. **(A)** *L. plantarum* was added at day 0 to the microbiota to reach 10^8^ to 10^9^ CFU/ml in TRs. The y-axis represents *L. plantarum* viable cell counts per ml reactor effluent. **(B)** Average carrying capacity for *L. plantarum* in different cultivated microbiota, whereas each data point represents one TR. Black: *L. plantarum* NZ3400B; red: *L. plantarum* IA10; blue: *L. plantarum* ΔLP_RS14990; orange: *L. plantarum* PA1.2_01 and NZ3400B; yellow: *L. plantarum* PA2_06 and NZ3400B. •: Adult 1; ■: Adult 2; ▴: Adult 3.a; ▾: Adult 3.b; ♢: Adult 4.

To investigate the ability of *L. plantarum* to colonize human adult microbiota in the PolyFermS model, it was added to five *in vitro* colonic microbiota. Since the number of total bacteria in different fermentations was not significantly different (data not shown), absolute *L. plantarum* cell counts could be used to compare colonization levels between different microbiota. *L. plantarum* cell counts were omitted. *L. plantarum* colonized all *in vitro* microbiota, reaching stable levels after ~5 days (Figure 2A). *L. plantarum* colonized the colonic microbiota of donor 2, 3.a, and 3.b at similar levels between 9^*^10^4^ and 2^*^10^5^ CFU/ml, yet at lower levels for donor 1 and 4, with respective stabilized concentrations of 2^*^10^3^ and 7^*^10^3^ CFU/ml (Figure 2A). Next, colonization of different *L. plantarum* strains were compared to NZ3400B. Strain IA10 colonized colonic microbiota of donor 2 at 2.4^*^10^5^ CFU/ml, very similar to NZ3400B at 1.8^*^10^5^ and 1.2^*^10^5^ CFU/ml (Figure 2B). Alike, ΔLP_RS14990 colonized the microbiota of donor 3 at 1.5^*^10^5^ and 2.7^*^10^5^ CFU/ml, respectively, and NZ3400B at 1.6^*^10^5^ and 1.8^*^10^5^ CFU/ml, respectively (Figure 2B). Finally, two strains were simultaneously added to the microbiota. Supplementation of *L. plantarum* PA1.2_01 and NZ3400B led to colonization at 1.6^*^10^5^ and 8.9^*^10^4^ CFU/ml, whereas PA2_06 and NZ3400B colonized lower at 5^*^10^4^ and 6^*^10^4^ CFU/ml (Figure 2B). Altogether, these data demonstrate the versatility of *L. plantarum* to colonize distinct microbiota communities, whereas colonization levels were microbiota-dependent.

### *L. plantarum* Organizes Itself Spatially in Colonic Microbiota

Colonization of the microbiota by *L. plantarum* could be promoted by biofilm formation. *L. plantarum* was detected in all biofilms formed in supplemented TRs at different abundances, depending on the donor microbiota and between parallel-operated reactors of the same model. *L. plantarum* counts in the total harvested biofilm from different TRs ranged between 10^8^ and 10^9^ CFU for donor 1, 10^3^ and 10^6^ CFU for donor 2, 10^6^ and 10^9^ CFU for donor 3.b, and 4^*^10^5^-7^*^10^5^ CFU for donor 4 models (Supplementary Figure S1). *L. plantarum* was thus established in both, the planktonic and sessile microbiota populations. Remarkably, when *L. plantarum* was in high abundance in the biofilm, it was in low abundance in the effluent. This negative correlation might suggest a direct link between biofilms and the planktonic *L. plantarum* fraction in the reactor.

### *L. plantarum* Colonization Levels Are Independent of the Introduction Dose

**Figure 3:**
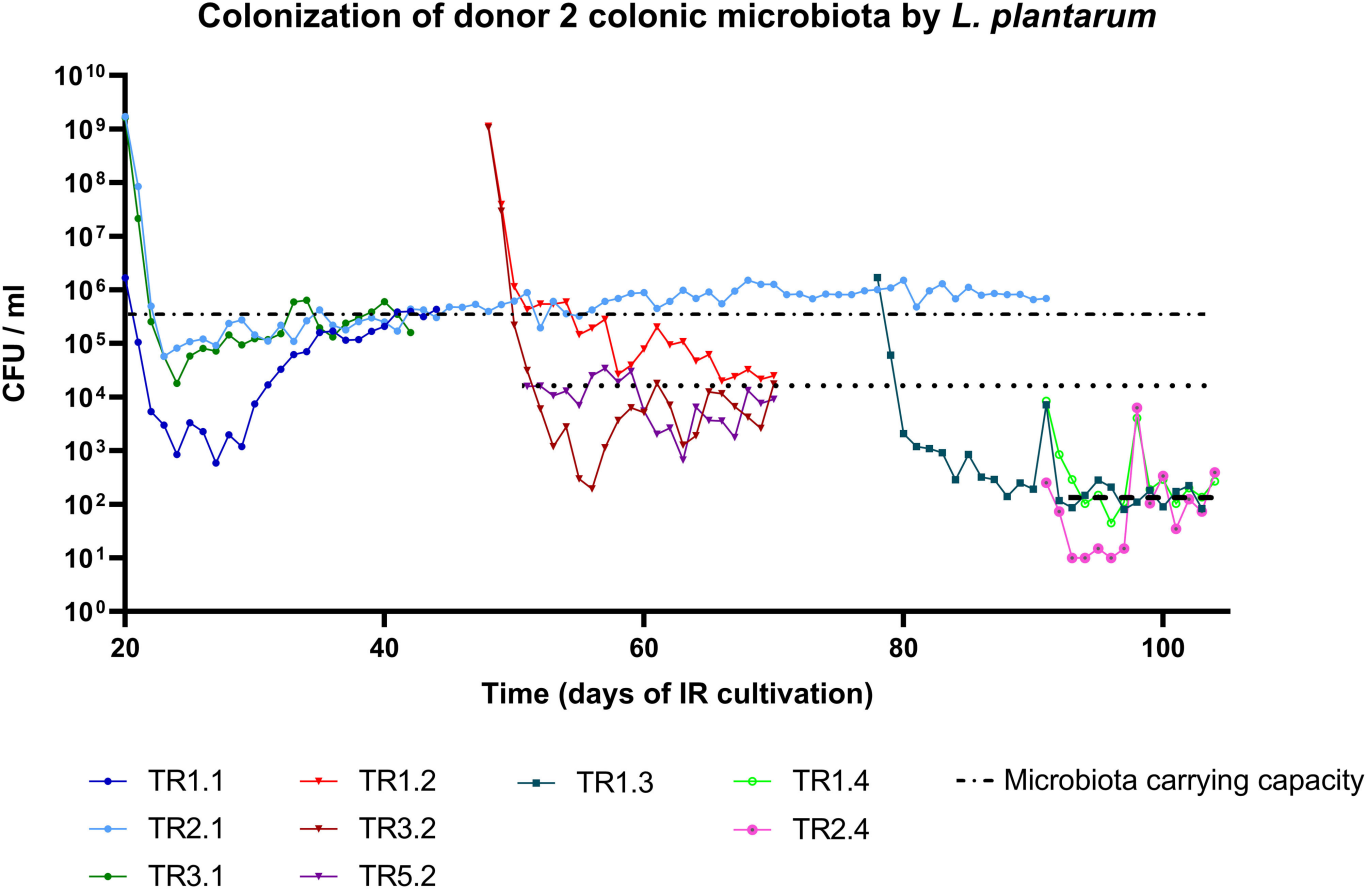
Change in carrying capacity of *in vitro* colonic microbiota of donor 2 over time of IR microbiota cultivation. The x-axis depicts the age of the colonic microbiota in the IR containing colonic microbiota of donor 2 and the y-axis represents *L. plantarum* viable cell count per ml reactor effluent. The dashed lines indicate the changing carrying capacity for *L. plantarum* with the age of the colonic microbiota.

**Figure 4:**
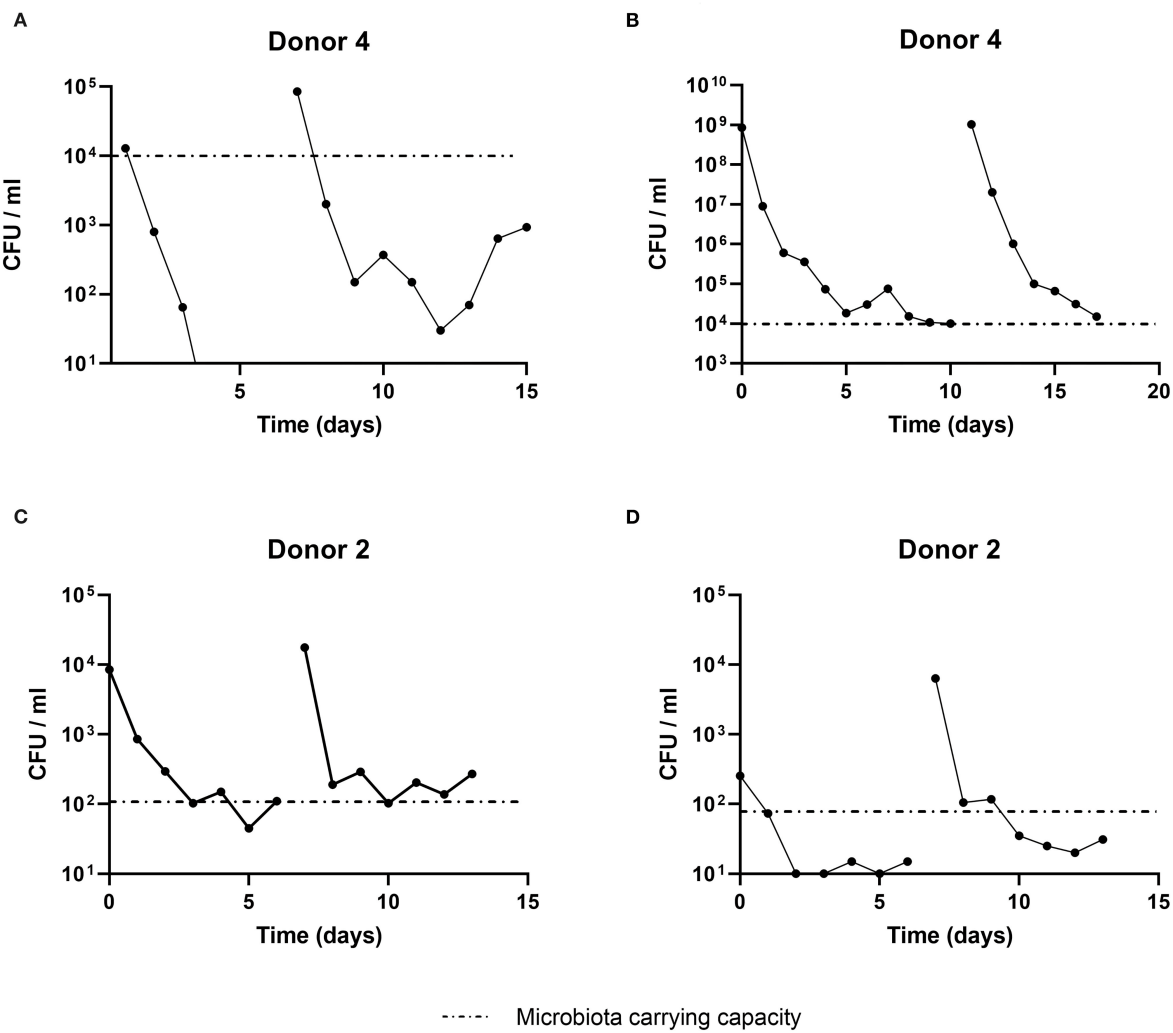
Effect of a second *L. plantarum* introduction dose on final colonization level. *L. plantarum* was added a second time to TR7 **(A)** and TR2 **(B)** containing colonic microbiota of donor 4 at days 7 and 11, respectively. Similarly, *L. plantarum* was added to TR1.4 **(C)** and TR2.4 **(D)** containing microbiota of donor 2 at days 0 and day 7. The y-axis represents *L. plantarum* viable cell counts per ml reactor effluent. The dashed line represents the present observed carrying capacity for *L. plantarum* in the corresponding colonic microbiota which is equal to the *L. plantarum* colonization level observed in parallel-operated TRs.

Next, we evaluated the effect of *L. plantarum* inoculation dose on colonization in the microbiota of donor 2 and 4 models that varied in their carrying capacity. The addition of 10^9^ and 10^6^ CFU/ml in donor 2 microbiota (TR1.1, TR2.1) resulted in colonization levels at 4^*^10^5^ CFU/ml after 41 days (Figure 3). In addition, inoculation of 10^9^ and 10^4^ (TR1.2, TR5.2) during a second treatment period led to similar colonization levels of 3^*^10^4^ and 1^*^10^4^ CFU/ml, respectively, and supplementation of 10^4^ and 10^2^ CFU/ml (TR1.4, TR2.4) during the fourth treatment period resulted again in similar colonization levels at 2^*^10^2^ CFU/ml (Figure 3). This shows that colonization levels are dose-independent. For donor 4 microbiota (TR7), *L. plantarum* was not able to colonize when supplemented at 10^4^ CFU/ml, a dose that corresponds to the microbiota carrying capacity (Figure 4A). Yet, a dose of 10^5^ CFU/ml resulted in successful colonization (Figure 4A). Thus, colonization may only be successful when the dose is equal to or above the microbiota carrying capacity for *L. plantarum*. Further, a second *L. plantarum* supplementation did not increase colonization levels (Figures 4B–D). Altogether, colonization success might be influenced by the dose, but *L. plantarum* colonization levels are independent of both, dose and repeated inoculation, in two distinct colonic microbiota.

### Human Adult Microbiota Composition and Metabolites Determine the Carrying Capacity for *L. plantarum*

In the next step, we analyzed the metabolic profile and the composition of all microbiota, based on 16S rRNA sequencing. There was no significant correlation between the carrying capacity for *L. plantarum* and the microbiota alpha diversity metrics richness and evenness (data not shown). On the other hand, beta diversity differed in IR microbiota. Microbiota with low carrying capacity were separated from microbiota with high carrying capacity in weighted beta diversity metrics (Supplementary Figure S2). Since weighted metrics incorporate taxa abundance and not only presence or absence, this suggests that the carrying capacity is influenced by similar microbiota structure.

**Figure 5:**
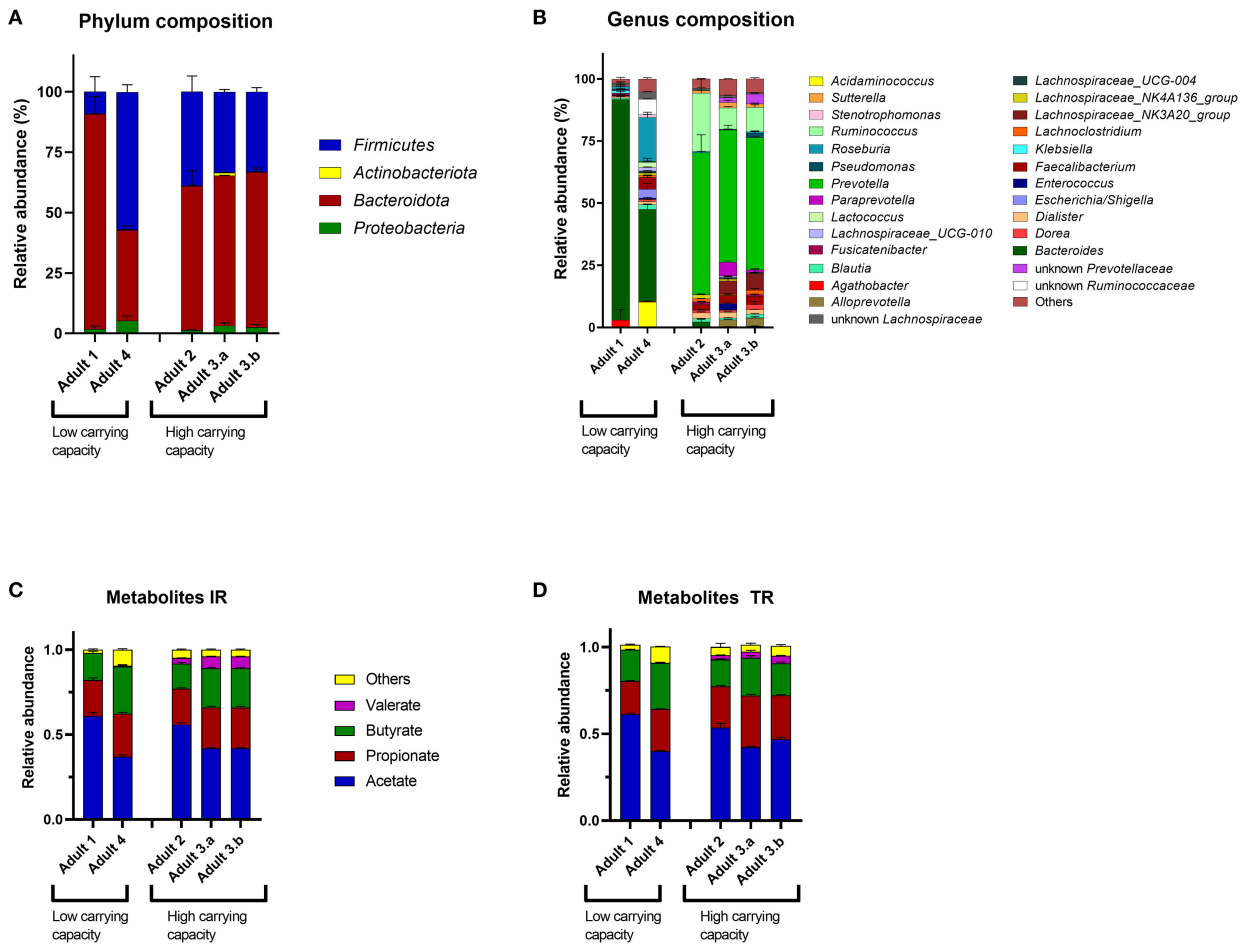
Microbiota composition and metabolite profile of different *in vitro* adult colonic microbiota. Relative abundance based on analysis of the 16S rRNA gene sequencing at **(A)** phylum and **(B)** genus level of 3 consecutive days of IRs containing colonic microbiota of donor 1 to 4. Values at genus level <1% are summarized in “Others.” The metabolic profile was analyzed in the **(C)** IR and the **(D)** corresponding TR for 3 consecutive days. The metabolites succinate, lactate, formate, iso-butyrate, and iso-valerate are summarized in “Others.” High carrying capacity indicates 10^5^ and low between 10^3^ and 10^4^ CFU *L. plantarum*/ml effluent.

Microbiota with higher carrying capacity were similar in composition on phylum and genus level in the IR (Figures 5A,B) and were dominated by *Prevotella* and *Ruminococcus*. Microbiota with lower carrying capacity mainly contained *Bacteroides* (Figure 5B). To further investigate the relationship between microbiota composition and the difference in carrying capacity, samples from 3 consecutive days with stable *L. plantarum* colonization levels of one TR per donor microbiota were selected for correlation analysis between ASVs and carrying capacity. The abundance of *Prevotella* was significantly positively (*r* = 0.76, *p* = 0.001) and of *Bacteroides* significantly negatively (*r* = −0.67, *p* = 0.007) correlated with the carrying capacity for *L. plantarum* (Supplementary Figure S3A). The *Prevotella* correlation was driven by *Prevotella* ASV0442, ASV0441, and *Prevotella copri* ASV0439 and the *Bacteroides* correlation by *Bacteroides vulgatus* ASV0475 and *Bacteroides uniformis* ASV0322 (Supplementary Figure S3B).

Microbiota differentiation according to carrying capacity was also reflected in the metabolic profile in the IR and TRs of the corresponding microbiota (Figures 5C,D). Microbiota in TRs of donor 2, 3.a, and 3.b were propiogenic (propionate:butyrate = 1.54 ± 0.05, 1.36 ± 0.04, and 1.38 ± 0.03, respectively), whereas microbiota of donor 1 and 4 exhibited similar concentrations of butyrate and propionate (propionate:butyrate = 1.06 ± 0.00 and 0.91± 0.01, respectively). Interestingly, microbiota with higher carrying capacity were characterized by higher valerate concentrations with 4.9 ± 0.2, 10.7 ± 0.1, and 10.6 ± 0.1 mM for microbiota of donor 2, 3.a, and 3.b, respectively, compared to the microbiota of donor 1 with no detected valerate and donor 2 with only 0.6 ± 0.02 mM valerate (Figures 5C,D). Analyzing TRs of all different adult microbiota for their carrying capacity and metabolic profile, the carrying capacity for *L. plantarum* correlated negatively with butyrate (*p* = 3^*^10^−9^) and positively with acetate (*p* = 6^*^10^−11^) and valerate (*p* = 2^*^10^−12^) (Supplementary Figure S4A). In summary, our data suggest that microbiota with alike composition and fermentation metabolic profile are colonized to similar carrying capacities by *L. plantarum*.

### Microbiota Maturity Influences the Carrying Capacity for *L. plantarum*

Microbiota of donor 2 was continuously cultivated in an IR for 104 days and TRs were connected at different time points, referred to as treatment periods in Figure 1. *L. plantarum* was added to TRs inoculated with microbiota from IR after 20, 48, 78, and 91 days of operation. When supplemented with a 20-day-old microbiota, *L. plantarum* stably colonized the microbiota for 70 days (TR 2.1). However, the carrying capacity for *L. plantarum* decreased with the time of cultivation, the microbiota maturity, from 10^5^ to 10^6^ CFU/ml, when *L. plantarum* was added to the 20 day-old microbiota, to 10^2^ CFU/ml, when added to the 91 day-old microbiota (Figure 3).

Microbiota maturity in the IR correlated positively with alpha diversity (observed species: *r* = 0.76, *p* = 8^*^10^−7^; Shannon diversity index: *r* = 0.65, *p* = 4^*^10^−4^; Pielou evenness: *r* = 0.48, *p* = 1^*^10^−2^). The observed species cannot increase over time in a reactor; hence, this indicates that the species grow above the detection limit. TRs that were connected to the IR at different time points were analyzed during stabilization (3 consecutive days before *L. plantarum* supplementation) and during colonization (3 consecutive days of stable *L. plantarum* counts). For the stabilization, the average carrying capacity of the corresponding TR was used for correlation analysis. The carrying capacity correlated negatively with alpha diversity during the microbiota stabilization (observed species: *r* = −0.68, *p* = 0.01; Shannon index: *r* = −0.51, *p* = 0.01; Pielou evenness: *r* = −0.52, *p* = 0.04) and also negatively with observed species (*p* = 1^*^10^−3^, *r* = −0.62) during *L. plantarum* colonization. These results suggest a higher colonization resistance toward an exogenous strain with increased richness, diversity, and evenness of the community.

Beta diversity analysis in TRs during stabilization revealed that the carrying capacity for *L. plantarum* was a significant contributing variable explaining composition differences for weighted and unweighted Jaccard (*p* = 0.001, *R*^2^ = 0.193 and *p* = 0.002, *R*^2^ = 0.14, respectively) and unweighted Unifrac (*p* = 0.001, *R*^2^ = 0.209) using PERMANOVA analysis. Similarly, the carrying capacity was a distinguishable variable in the colonization period in unweighted Jaccard (*p* = 0.001, *R*^2^ = 0.104) and unweighted Unifrac (*p* = 0.003, *R*^2^ = 0.112). In contrast to the comparison above between different microbiota, where the carrying capacity is influenced by similar microbiota structure, it seems that during colonization of the same microbiota, carrying capacity might depend more on microbiota composition, meaning the absence or presence of taxa. This is in accordance with the higher richness in low carrying capacity microbiota.

### Valerate Concentration Correlates With the Change in Carrying Capacity for *L. plantarum*

The carrying capacity correlated negatively with all SCFAs concentrations in TRs containing microbiota of donor 2 during stabilization, that is, before *L. plantarum* supplementation (Supplementary Figure S4B). The same pattern was observed during colonization, that is, time with stable *L. plantarum* colonization, except for valerate, which correlated positively. This suggests a role of valerate in microbiota carrying capacity for *L. plantarum* (Supplementary Figure S4C; *r* = 0.41, *p* = 10^−10^). A detailed view of the cultivated microbiota of donor 2 revealed that many ASVs correlated with both, carrying capacity and valerate concentration, although not always significant, suggesting that the valerate-producing capacity of the microbiota might influence the carrying capacity for *L. plantarum* (Supplementary Figure S5). This is in accordance with the above-described higher valerate concentrations in microbiota exhibiting higher carrying capacity (Figures 5C,D) and the positive correlation between valerate concentration on carrying capacity across all microbiota (Supplementary Figure S4A). Altogether, this suggests a putative role of valerate in the microbiota carrying capacity for *L. plantarum*.

### Microbiota Carrying Capacity for *L. plantarum* Can Be Modulated by Valerate

**Figure 6:**
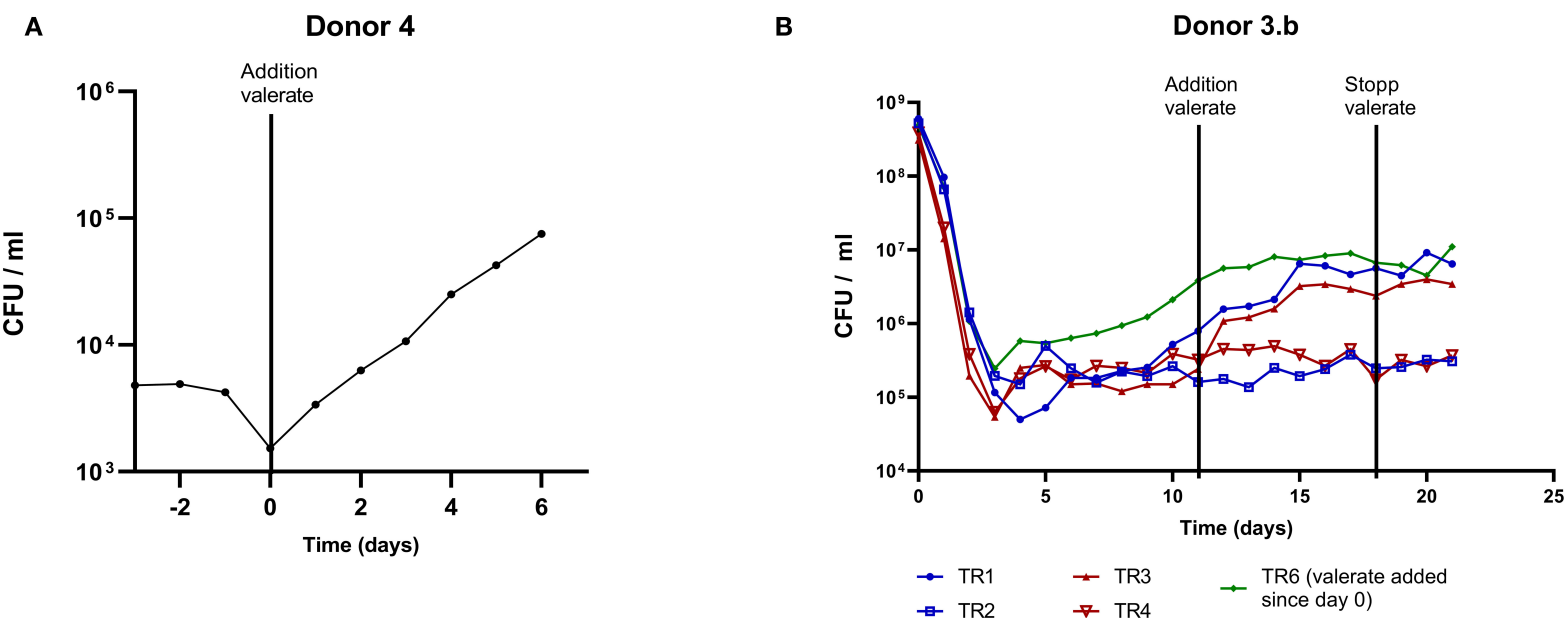
Modulation of the human adult microbiota carrying capacity for *L. plantarum* by valerate supplementation. Valerate was continuously supplemented after stable *L. plantarum* microbiota colonization into **(A)** TR1 containing microbiota of donor 4 and **(B)** TR1, TR3, and TR6 containing colonic microbiota of donor 3.b, whereas valerate was continuously added to TR6 already since reactor connection. TR2 and TR4 served as control reactors. The x-axis depicts the time of *L. plantarum* cultivation and the y-axis represents *L. plantarum* viable cell count per ml reactor effluent. The solid line indicates the start and stop of valerate supplementation. Blue represents *L. plantarum* NZ3400B and red ΔLP_RS14990.

Valerate was investigated as a modulator for the carrying capacity for *L. plantarum* in the microbiota of donor 4 and 3.b. After successful *L. plantarum* colonization, valerate was continuously added to donor 4 microbiota (TR1). The carrying capacity for *L. plantarum* increased by about 1.5 logs (Figure 6A) during 6 days of valerate supplementation, which confirms the role of valerate in *L. plantarum* carrying capacity. To validate this observation, valerate was continuously added after stable *L. plantarum* colonization to microbiota of donor 3.b (TR1, TR3), whereas TR2 and TR4 served as control reactors (Figure 1). *L. plantarum* increased one log during valerate supplementation and colonization levels were maintained upon the termination of valerate supplementation (Figure 6B). Further, the same *L. plantarum* colonization level was reached when supplementing valerate before *L. plantarum* inoculation (Figure 6B, TR6).

### Valerate Induces Microbiota-Dependent Changes in the Composition

Analyses of microbiota composition and metabolites revealed that valerate supplementation did neither lead to significant changes in microbial metabolites, except for valerate, nor microbiota alpha diversity. PERMANOVA analysis of 3 days before and all days during valerate supplementation compared to the control reactors revealed that differences in valerate concentrations partially explained differences in weighted Jaccard (*R*^2^ = 0.139, *p* = 0.001) and weighted Unifrac (*R*^2^ = 0.108, *p* = 0.013). Influencing both, weighted and unweighted metrics, indicates that the valerate induced changes in community composition and structure.

Valerate led to microbiota-dependent changes in composition (Supplementary Figures S6A,B). Most pronounced was the increase in *Enterococcaceae* in the microbiota of donor 3.b, which was not observed when supplementing valerate upon reactor connection (TR6). Valerate induced a prolonged shift in the community after interrupting the supplementation (Supplementary Figure S6B). This is in agreement with the observation that the carrying capacity for *L. plantarum* remained unchanged after valerate supplementation termination. To investigate changes in the ASV level, DESeq analysis of 3 days before valerate supplementation and all days during valerate supplementation was performed (Supplementary Table S1). In microbiota of donor 4, valerate led to a significant increase in *Veillonellaceae* and a decrease in *Escherichia*/*Shigella* ASVs. All other ASV changes were not consistent per family nor genus. In microbiota of donor 3.b, valerate led to a consistent significant increase in *Enterococcus* and *Veillonellaceae* ASVs, whereas changes in *Lachnospiraceae, Oscillospiraceae, Prevotellaceae, Ruminococcaceae*, and *Faecalibacterium prausnitzii* were ASV-dependent. Although valerate modulated the carrying capacity in a microbiota-independent manner, different ASVs were impacted within and across microbiota. This makes it difficult to pinpoint single ASVs responsible for the increase in microbiota carrying capacity for *L. plantarum*. However, some ASVs that significantly increased during valerate treatment (Supplementary Table S1) were already positively correlated with microbiota carrying capacity (Supplementary Figure S3): *Suterella* (ASV0031), *Prevotella* (ASV0441), and *Prevotella copri*. Further, *Lachnospira* (ASV1226) increased during valerate treatment in the colonic microbiota of donor 3.b and 4 and was already seen to be positively correlated with valerate concentration in TRs containing microbiota of donor 2 (Supplementary Figure S5).

## Discussion

Humans ingest large amounts of bacteria through food, beverages, or probiotic supplements (Lang et al., 2014). It is of interest to assess how these bacteria establish in and interact with the resident gut community. The interplay between host factors, the microbiota, and the exogenous strain makes it difficult to assign observed effects to specific interactions. *In vitro* intestinal models have the advantage to exclude the host-microbe interactions and thus enable to solely focus on microbe-microbe interactions. Therefore, we used the continuous gut fermentation model PolyFermS to investigate the colonization process of *L. plantarum* NZ3400 into human adult microbiota, a derivative of the probiotic strain WCFS1, and to identify related community features. We showed that *L. plantarum* was able to colonize all different human adult microbiota and that it organized itself spatially in the planktonic and sessile population of the *in vitro* colonic microbiota. Moreover, we showed previously that *L. plantarum* strains that were isolated from PolyFermS colonic microbiota exhibited an auto-aggregation phenotype which may contribute as another way of the spatial organization by *L. plantarum* in this kind of microbiota (Isenring et al., 2021a). This might assist successful colonization by providing new niche opportunities (Chesson, 2000a). In contrast to the colonization success observed in this *in vitro* study, it was previously shown that *L. plantarum* is a rather poor colonizer of the *in vivo* human gut microbiota (Vesa et al., 2000; Costa et al., 2014). This difference might be explained by the additional host factors in *in vivo* experiments and the mode of supplementation. Further, we showed that colonization levels were not manipulable by the introduction dose or second administration and that the introduction dose only influenced colonization success when it was below the carrying capacity of the resident community. Although these results are preliminary and would need further validation, our data suggest that biotic interactions and characteristics of the resident community are more important than introduction parameters.

The carrying capacity for *L. plantarum* was microbiota-dependent and microbiota with higher carrying capacity were similar in composition and metabolic profile. Colonization success of an exogenous strain was previously shown to be influenced by the resident colonic microbiota (Frese et al., 2012; Maldonado-Gomez et al., 2016). Further, the carrying capacity largely depends on resource availability and species competition to efficiently utilize the resources (Shea and Chesson, 2002; Bray et al., 2019; Contijoch et al., 2019). In this study, resources fed into the system by continuous medium inflow were always the same. However, the differences in microbiota composition can lead to different resource utilization and production and thus overall availability for other bacteria.

The microbiota carrying capacity for *L. plantarum* decreased during 3 months of continuous cultivation. Microbiota maturation was characterized by higher community richness and evenness and a higher colonization resistance toward *L. plantarum*. This was only investigated for one microbiota and would therefore need repetition for validation. However, our results are in accordance with observations that ecological niches and thus carrying capacity can vary in time and space (Chesson, 2000b; Shea and Chesson, 2002; Bauer et al., 2018; Wienand et al., 2018). Less mature communities have less time for optimal adaptation and therefore fewer species to fill all available niches, which results in lower colonization resistance (Shea and Chesson, 2002). In accordance with our results, higher colonization resistance was linked to higher diversity and evenness because resources are more efficiently exploited and free niches are diminishing (Shea and Chesson, 2002; Dunstan and Johnson, 2006; Jousset et al., 2011; Kinnunen et al., 2016).

The carrying capacity of different microbiota within donor 2 experiments correlated positively with valerate concentration. Thus, *L. plantarum* either leads to an increase in valerate production or valerate enables *L. plantarum* to increase in abundance. Increased valerate concentrations were also observed after *L. plantarum* addition in broilers (Han et al., 2018). However, no increase in valerate concentration was observed in our study when *L. plantarum* abundance increased in the reactor (data not shown). Furthermore, the microbiota of donors with higher carrying capacity inherently exhibited higher valerate concentrations and were dominated by the *Prevotella* genus. This is in line with previous *in vivo* data showing that microbiota of healthy adults exhibited higher fecal valerate concentrations with a higher prevalence of *Prevotella* and *Dorea* and lower fecal valerate concentrations with a high prevalence of *Bacteroides* (Tap et al., 2015). Further, *Prevotella* species such as *Prevotella copri* and *Prevotella stercorea* belong to the few-known valerate producers (Hayashi et al., 2007; Almeida et al., 2019).

SCFAs like valerate can inhibit bacterial growth and influence gene expression (Lamas et al., 2019). Indeed, *L. plantarum* growth in valerate supplemented MRS was impaired at 12 mM (data not shown). Valerate inhibits *Clostridium difficile in vitro* and *in vivo* and the inhibition is strain and species-specific (McDonald et al., 2018). It is therefore likely that valerate negatively affects colonic microbiota members that compete with *L. plantarum*. Despite the microbiota-independent increase in carrying capacity induced by valerate supplementation, changes in the ASV level were microbiota-dependent, suggesting that there are several niche opportunities for *L. plantarum* within one microbiota. This is probably based on functional redundancy where several taxa are interchangeable due to shared metabolism.

In conclusion, we demonstrated the suitability of the PolyFermS *in vitro* gut model to investigate the interaction between an exogenous strain and distinct *in vitro* colonic microbiota with the advantage of excluding the host and strictly controlling for external and dietary factors. We were able to show that the investigation of the colonization process and following colonizer-microbiota interactions allows the identification of carrying capacity modulators. Gained knowledge about mechanisms involved in the colonization of exogenous strains will further enable improvements in microbiome manipulation and probiotic application strategies. In addition, this approach can be used to elucidate ecological dynamics, probiotic and pathogen colonization processes, and carrying capacity modulators for disease prevention. However, results should be evaluated in *in vivo* studies to assess to which extent they can be translated to humans.

## Data Availability Statement

The datasets presented in this study can be found in online repositories. The names of the repository/repositories and accession number(s) can be found at: https://www.ebi.ac.uk/ena, PRJEB44549.

## Author Contributions

JI, CJ, MS, CL, and AG designed the study. Experiments were executed and data were analyzed by JI. The manuscript was drafted by JI and AG and critically reviewed by all authors. All authors contributed to the article and approved the submitted version.

## Funding

This study was funded by the ETH Zürich Research Grant Program (ETH-42 16-1). Open access funding provided by ETH Zürich.

## Conflict of Interest

The authors declare that the research was conducted in the absence of any commercial or financial relationships that could be construed as a potential conflict of interest.

## Publisher's Note

All claims expressed in this article are solely those of the authors and do not necessarily represent those of their affiliated organizations, or those of the publisher, the editors and the reviewers. Any product that may be evaluated in this article, or claim that may be made by its manufacturer, is not guaranteed or endorsed by the publisher.
