# Supplementary material for: Identification of Valerate as Carrying Capacity Modulator by Analyzing Lactiplantibacillus plantarum Colonization of Colonic Microbiota in vitro
Source: Front Microbiol. 2022 May 30;13:910609. doi: 10.3389/fmicb.2022.910609 (PMC9197689; doi:10.3389/fmicb.2022.910609)
Supplement: Supplementary file 1 [file Data_Sheet_1.docx]

# **Supplementary Material**


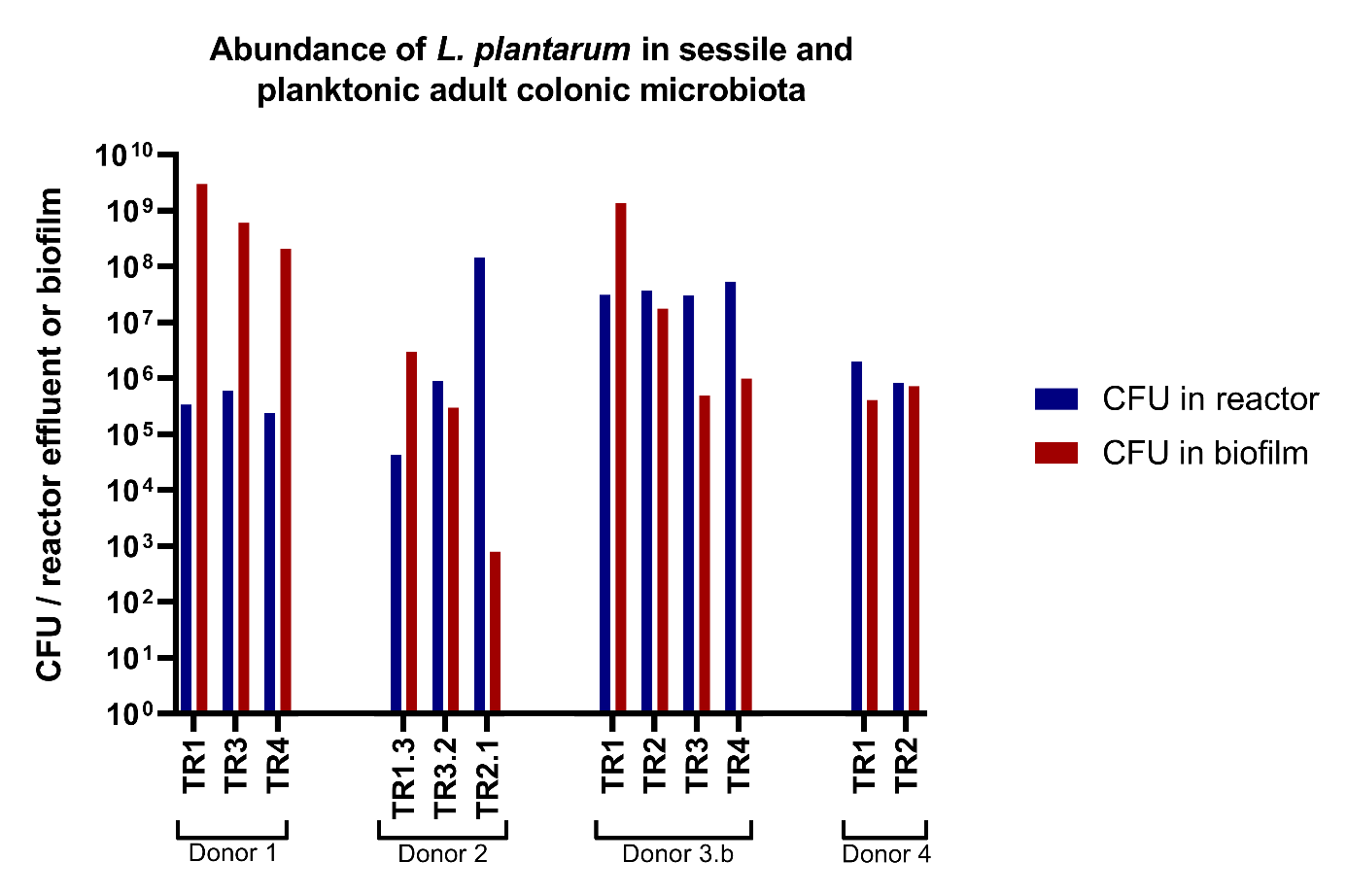


**Supplementary Figure S1**. *L. plantarum* abundance in reactor effluent and biofilm of different *in vitro* adult colonic microbiota. The y-axis depicts *L. plantarum* viable cell counts.


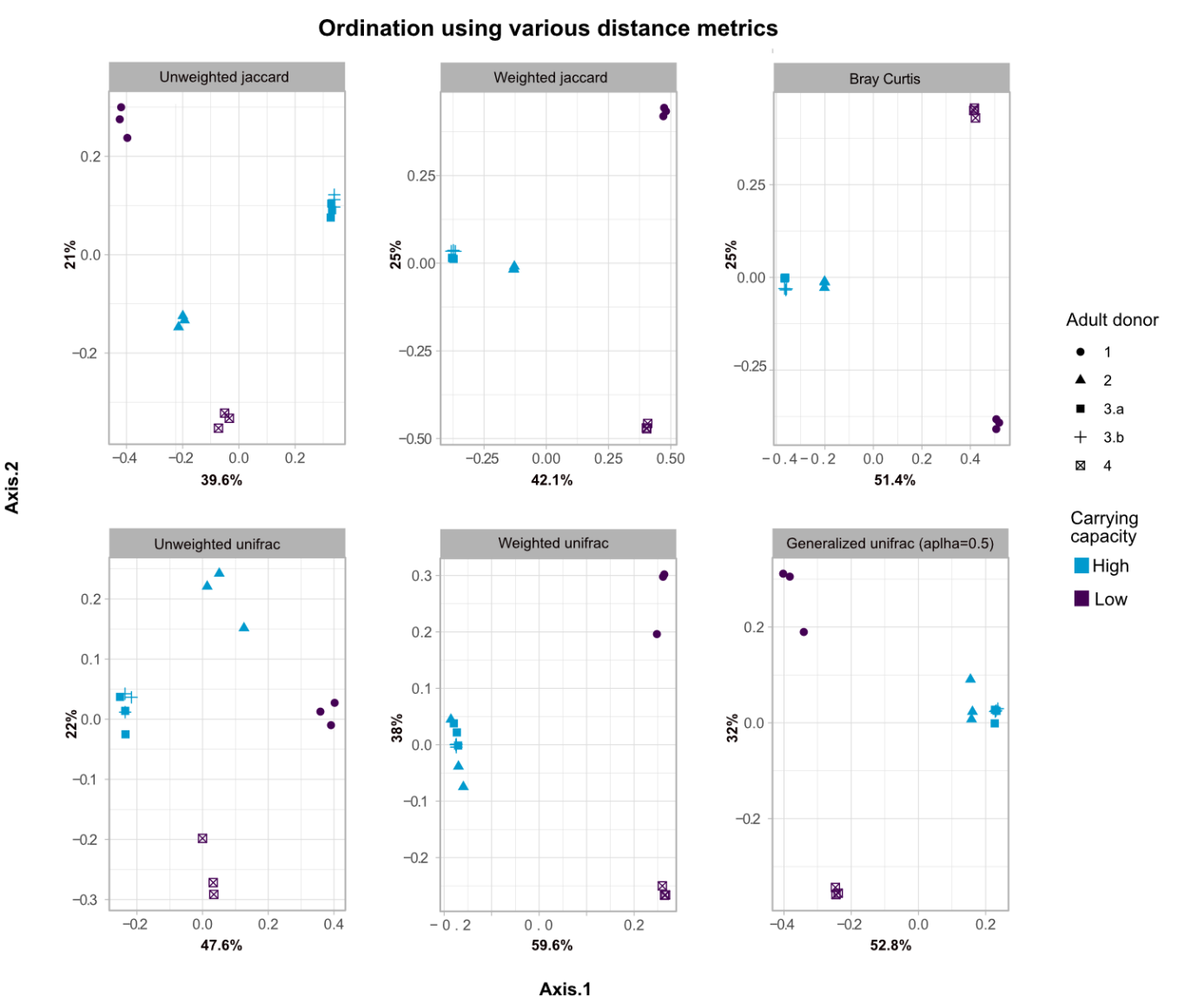


**Supplementary Figure S2**. Beta diversity metrics of different human adult *in vitro* colonic microbiota. For each donor, samples from the corresponding IR at three consecutive days after two weeks of cultivation were analyzed. Colors divides the donors according to their carrying capacity, with donor 2, 3.a and 3.b having a higher (10^5^ CFU/ml) and donor 1 and 4 a lower (10^3^-10^4^ CFU/ml) carrying capacity for *L. plantarum*.


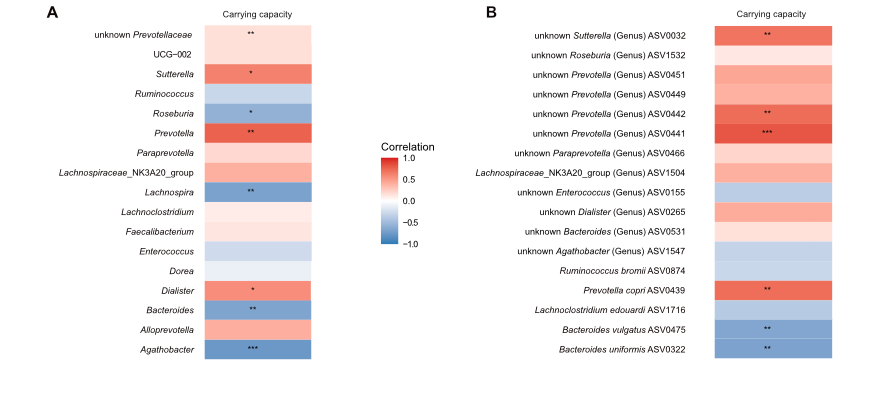


**Supplementary Figure S****3**. Microbiota taxa correlation with carrying capacity for *L. plantarum* across all human adult *in vitro* microbiota. For each donor, three consecutive days with stable *L. plantarum* colonization levels and no additional treatment of one TR were chosen. Correlation analysis was done on **(A)** genus and **(B)** ASV level. Red indicates positive and blue negative correlations with gut microbiota carrying capacity whereas the significance level is given by the stars: *: p≤0.05, **: p≤0.01 and ***: p≤0.001.


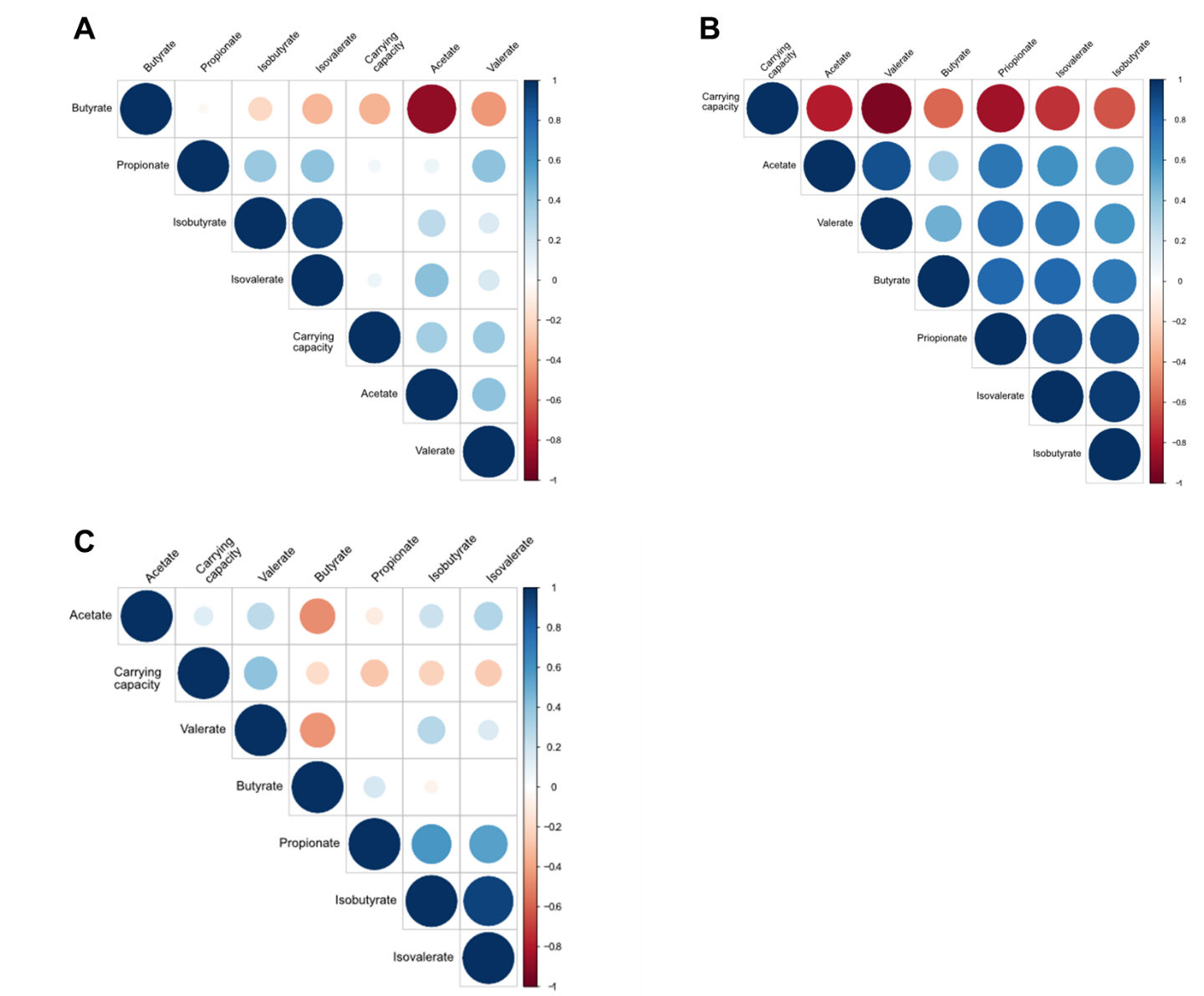


**Supplementary Figure S4.** Correlation between metabolite concentration and adult gut microbiota carrying capacity for *L. plantarum*. **(A)** Samples from TRs inoculated with different adult microbiota (n = 5, total analyzed samples = 324) were pooled and metabolite concentration was correlated with the carrying capacity. **(B)** Samples from all TRs containing colonic microbiota of donor 2 during stabilization (three consecutive days before *L. plantarum* supplementation) were pooled for correlation analysis (n = 24). **(C)** Samples from TRs containing gut microbiota of donor 2 (n = 299) were pooled for analysis to detect correlation after *L. plantarum* supplementation. For **(A)** and **(C)** only samples after six days upon *L. plantarum* supplementation were considered when valerate and *L. plantarum* colonization levels were stable. Red color indicates negative and blue positive correlation of the corresponding SCFA with the gut microbiota carrying capacity for *L. plantarum*. Only significant correlations (p≤0.05) are depicted, whereas the circle size corresponds to increased significance.


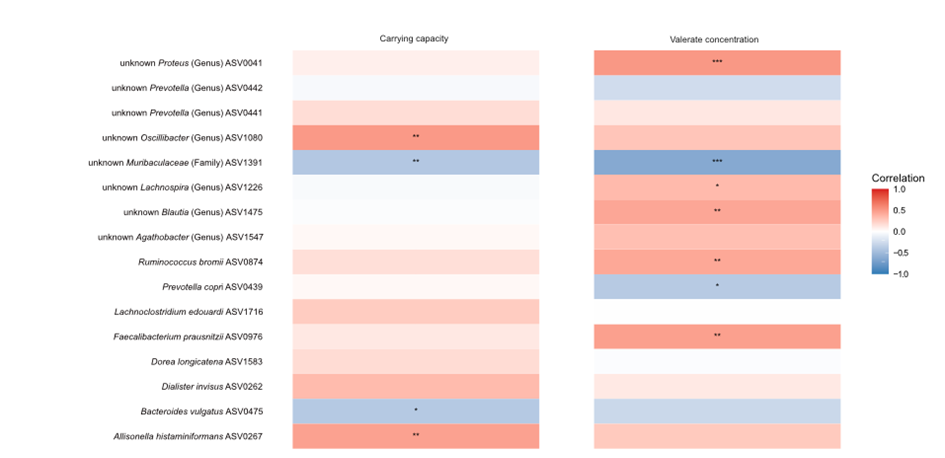


**Supplementary Figure S5.** Microbiota ASV correlation with carrying capacity for *L. plantarum* and valerate concentration of TRs (n=7, n(days)=44) containing colonic microbiota of donor 2. Red indicates positive and blue negative whereas the significance level is given by the stars: *: p≤0.05, **: p≤0.01 and ***: p≤0.001.


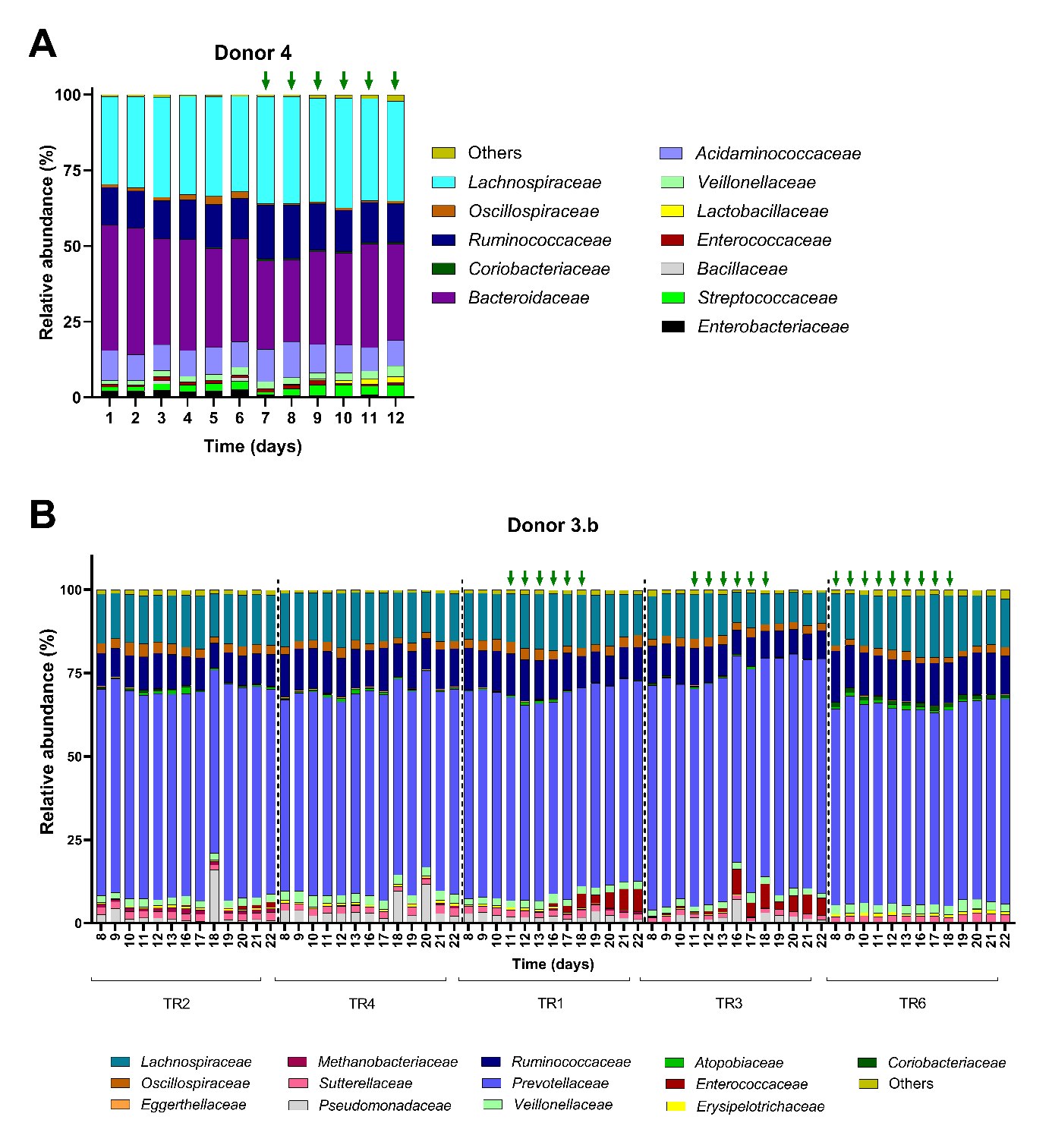


**Supplementary Figure S6.** Changes on family level gut microbiota composition before, during and after valerate supplementation. **(A)** Shows six days before and during valerate supplementation in TR1 containing gut microbiota of donor 4. **(B)** Valerate was supplemented into TR1 and TR3 containing gut microbiota of donor 3.b whereas TR2 and TR4 served as control reactor. Further, valerate was continuously added into TR6 since reactor connection. Green arrows indicate the days with continuous valerate supplementation.

Table S1: DESeq analysis of valerate treatment induced changes in microbial composition.

Comparison of three days before and all days during valerate supplementation within one TR. Only changes that were not observed in the control reactors were listed in this table. - : significantly (p≤0.05) decreased after valerate supplementation; + : significantly (p≤0.05) increased after valerate supplementation.
